# Editorial: Molecular advances in diagnosis and treatment of CNS tumors, volume II

**DOI:** 10.3389/fonc.2023.1146805

**Published:** 2023-01-30

**Authors:** Clark Chen, Liam Chen

**Affiliations:** ^1^ Department of Neurosurgery, University of Minnesota Medical School, Minneapolis, MN, United States; ^2^ Department of Pathology, University of Minnesota Medical School, Minneapolis, MN, United States

**Keywords:** brain tumor, glioblastoma, glioma, astrocytic, embryonal

It’s been barely two years since the publication of the first volume of the Research Topic “*Molecular advances in diagnosis and treatment of CNS tumors*” (Zhang et al.). In the last two years, researchers across the world continue to make great strides in the fight against the brain and spinal cord tumors. Here we are delighted to present the second volume of this research topic which includes ten original research articles, four review articles and one meta-analysis that cover several important themes:

Some of the manuscripts published in this volume concern studies that further elucidate the molecular pathobiology of particular CNS tumors. For IDH-wildtype diffuse astrocytic tumors in adults, the fifth edition of the WHO classification of CNS tumors considers IDH-wild type lower-grade glioma (LGG) as a provisional entity as tumors that conform to this diagnosis most likely can be reclassified as other tumors with additional genetic analyses. Indeed, Wang et al. showed that the differentially expressed genes in LGG patients were mainly enriched in metabolic pathways and pathways in cancer and in the function of signal transduction and positive regulation of GTPase activity, whereas in glioblastoma (GBM) patients, they were mainly enriched in the PI3K-Akt signaling pathway and in the functions of apoptotic process and oxidation-reduction process. Wang et al. studied the role of cancer-derived immunoglobulin G (cancer-IgG) in glioma which has been known to be associated with several malignancies such as breast cancer, colorectal cancer, and lung cancer. They found that the expression of cancer-IgG was higher in glioma and molecular subtypes with poor prognosis. The overall survival of patients with a high expression of cancer-IgG was worse in the stratified analysis. In addition, cancer-IgG may function in phagosome, antigen processing and presentation, extracellular matrix structural constituent, antigen binding, and collagen-containing extracellular matrix. Traditionally, the histology-based glioma classification is composed of multiple different molecular subtypes with distinct behavior, prognosis, and response to therapy, and now each aspect can be assessed by corresponding emerging MR sequences like amide proton transfer-weighted MRI, inflow-based vascular-space-occupancy MRI, and radiomics informed algorithms. Indeed, Wei and Wei discussed these recent advances in MRI in their timely review to demonstrate that preoperative diagnosis of glioma has stepped into molecular and algorithm-assisted levels.

Epilepsy is a common clinical symptom in patients with glioma, which can impair neurocognitive function and quality of life. In the only meta-analysis study accepted in the second volume, Song et al. investigated the relationship between five commonly used tumor molecular markers and the incidence of perioperative epilepsy in patients with glioma. Their findings showed that isocitrate dehydrogenase 1 (IDH1) mutation was significantly correlated with the incidence of preoperative epilepsy, but not with intraoperative and postoperative epilepsy. There was no correlation between O6-methylguanine-DNA methyltransferase methylation and 1p/19q deletion and the incidence of perioperative epilepsy.

The rest of the accepted manuscripts studied other common CNS tumors. Liang et al. discovered that aurora kinase A and kinesin family member 20A may be involved in the initiation and development of medulloblastoma. Liu et al. studied the expression of tumor biomarkers and evaluate their clinical significance in non-functioning pituitary adenomas with different invasion patterns. Cathepsin K has the potential as a marker for sphenoid sinus invasion, whereas MMP9 and MMP2 may be markers for cavernous sinus invasion. Two comprehensive reviews, one by Zhang et al. on biomarkers associated with vestibular schwannoma growth, the other one by Ferguson et al. on the advances in leptomeningeal disease diagnosis with a focus on the role of circulating tumor DNA, have been included for readers’ interests.

While CNS tumors can now be much more precisely characterized than a few years ago, the translation of this increased knowledge into more effective treatments is still seriously lagging behind. Three studies explored the benefits of promising antitumor therapeutic agents. Tsai et al. analyzed the impact of valproic acid (VPA) on GBM patient survival and its possible correlation with temozolomide (TMZ) treatment and p53 gene mutation. Their analysis of clinical data indicates that the survival benefit of a combined TMZ and VPA treatment in GBM patients is dependent on their p53 gene status. In cellular experiments, VPA enhanced the antineoplastic effect of TMZ by enhancing p53 activation and promoting the expression of its downstream pro-apoptotic protein, PUMA. Thus GBM patients with wild-type p53 may benefit from a combined TMZ+VPA treatment. In their seminal review, Bonafé et al. presented mounting evidence to argue that the effectiveness of four natural plant compounds, namely caffeine, dipotassium glycyrrhizinate (DPG), curcumin, and euphol is likely achieved through enhancing apoptosis-related pathways and cell cycle impairment in GBM cell lines. Furthermore, antitumoral effect of these plant compounds on GBM cell lines through microRNAs (miRNAs) modulation was investigated. Interestingly, only DPG and curcumin were found to be effective on miRNA modulation. The same group further evaluated in a separate study the expression profiles of miRNAs related to NF-κB suppression in the T98G GBM cell line after DPG exposure. The most over-expressed miRNAs were miR-4443 and miR-3620. Their results suggest that DPG inhibits cell viability by activating apoptosis and inhibiting cell proliferation and stem cell subpopulation formation through miR-4443 and miR-3620 upregulation. Both miRNAs are responsible for the post-transcriptional inhibition of NF-κB by CD209 and TNC modulation.

One study focused on identifying novel therapeutic targets for glioma. Through analysis of The Cancer Genome Atlas (TCGA) data, Ma et al. identified that OLFML2A is a key promotor of gliomagenesis. OLFML2A expression was significantly upregulated in glioma specimens and positively correlated with pathological grades and shorter overall survival in glioma patients. Mechanistically, OLFML2A downregulation inhibits Wnt/β-catenin signaling by upregulating amyloid precursor protein expression and reducing stabilized β-catenin levels, leading to the repression of MYC, CD44, and CSKN2A2 expression. By uncovering the oncogenic effects in human and rodent gliomas, their data support OLFML2A as a potential therapeutic target for glioma.

Finally, this volume includes three studies investigating the independent prognostic markers in CNS tumor. Primary central nervous system diffuse large B-cell lymphoma (PCNS-DLBCL) is an uncommon non-Hodgkin lymphoma subtype, and its clinical and pathological characteristics remain unclear. Qi et al. retrospectively evaluated PCNS-DLBCL patient data to determine clinical and pathological characteristics and prognostic factors. Multivariate analysis identified Ki-67 and CD3 as independent prognostic factors for survival. Moreover, multifocal lesions and deep brain involvement were unfavorable independent prognostic markers for progression-free survival. This study indicates that targeted drug development for adverse prognostic factors is possible and provides guidance for clinical treatment decision-making. He et al. showed that higher prognostic nutrition index (PNI) was an independent prognostic factor for grade IV glioma. Interestingly, they found that the nomogram including preoperative PNI, age, extent of resection, number of gliomas, and MGMT methylation status could predict the prognosis of patients with grade IV glioma well. Wang et al. selected five DNA damage repair genes (DDRGs) including CDK4, HMGB2, WEE1, SMC3 and GADD45G to construct a DDRGs signature for glioma. The survival analysis showed that the DDRGs signature could differentiate the outcome of the low- and high-risk groups. The immune microenvironment analysis revealed that more immunosuppressive cells, such as tumor associated macrophages and regulatory T cells, were recruited in the high-risk group. Therefore, the five DDRGs signature and its impact on the infiltration of immunosuppressive cells could potentially predict the prognosis.

In summary, in the second volume of the Research Topic entitled “Molecular Advances in Diagnosis and Treatment of CNS Tumors” a tremendous amount of information with relevance for neurooncology has been published. Hopefully, this collection of knowledge can be exploited to help drastically improve the lives of patients that suffer from a CNS tumor.

## Author contributions

All authors listed have made a substantial, direct, and intellectual contribution to the work and approved it for publication.

